# Enzymatic and non-enzymatic isolation systems for adipose tissue-derived cells: current state of the art

**DOI:** 10.1186/s13619-015-0020-0

**Published:** 2015-09-30

**Authors:** Eleni Oberbauer, Carolin Steffenhagen, Christoph Wurzer, Christian Gabriel, Heinz Redl, Susanne Wolbank

**Affiliations:** 1 Ludwig Boltzmann Institute for Experimental and Clinical Traumatology, AUVA Research Center, Linz/Vienna, Austria; 2 Austrian Cluster for Tissue Regeneration, Vienna, Austria; 3 Red Cross Blood Transfusion Service of Upper Austria, Linz, Austria

**Keywords:** Human adipose tissue, Stromal vascular fraction, Adipose-derived stromal/stem cells, Enzymatic, Non-enzymatic, Isolation systems

## Abstract

In the past decade, adipose tissue became a highly interesting source of adult stem cells for plastic surgery and regenerative medicine. The isolated stromal vascular fraction (SVF) is a heterogeneous cell population including the adipose-derived stromal/stem cells (ASC), which showed regenerative potential in several clinical studies and trials. SVF should be provided in a safe and reproducible manner in accordance with current good manufacturing practices (cGMP). To ensure highest possible safety for patients, a precisely defined procedure with a high-quality control is required. Hence, an increasing number of adipose tissue-derived cell isolation systems have been developed. These systems aim for a closed, sterile, and safe isolation process limiting donor variations, risk for contaminations, and unpredictability of the cell material. To isolate SVF from adipose tissue, enzymes such as collagenase are used. Alternatively, in order to avoid enzymes, isolation systems using physical forces are available. Here, we provide an overview of known existing enzymatic and non-enzymatic adipose tissue-derived cell isolation systems, which are patented, published, or already on the market.

## Introduction

Subcutaneous human adipose tissue is an abundant source of mesenchymal stromal/stem cells (MSC), which are promising for several therapeutic applications in esthetic and regenerative medicine. For a long time, adipose tissue has been considered to be an inert tissue and has usually been discarded as surgical waste after liposuction [1, 2]. Nevertheless, it is a highly vascularized tissue and an abundant source of multiple cell types such as MSC [3, 4]. MSC with similar characteristics can be found in a variety of other tissues including the bone marrow, muscle, connective tissue, skin, placenta, blood, cord blood, synovium, periosteum, and perichondrium [2, 5–19]. Bone marrow-derived mesenchymal stromal/stem cells (BM-MSC) have been extensively used since their discovery in the early 70s [20], but harvesting bone marrow is accompanied by certain drawbacks including painful procurement as well as low stem cell yield. In comparison to BM-MSC, MSC from adipose tissue, the adipose-derived stromal/stem cells (ASC), occur at a 100–1000-fold higher frequency within adipose tissue on a volume basis [21]. Harvesting adipose tissue is minimally invasive and less painful, and liposuction is one of the most commonly performed cosmetic procedures without general anesthesia [1]. Additionally, the adipose source offers two options for selection of regenerative cells: the stromal vascular fraction (SVF) and the ASC contained therein.

### The stromal vascular fraction (SVF)

SVF is a heterogeneous mixture of cells isolated by enzymatic or non-enzymatic dissociation of adipose tissue followed by centrifugation in order to remove the differentiated adipocytes, which float over the aqueous layer. The heterogeneous population of SVF includes endothelial cells, erythrocytes, fibroblasts, lymphocytes, monocytes/macrophages, and pericytes among others, but most importantly ASC [22–26]. SVF may be used as a source material to isolate cells for tissue regeneration and has already been applied in clinical trials [27–32].

### Adipose-derived stromal/stem cells (ASC)

**Fig. 1:**
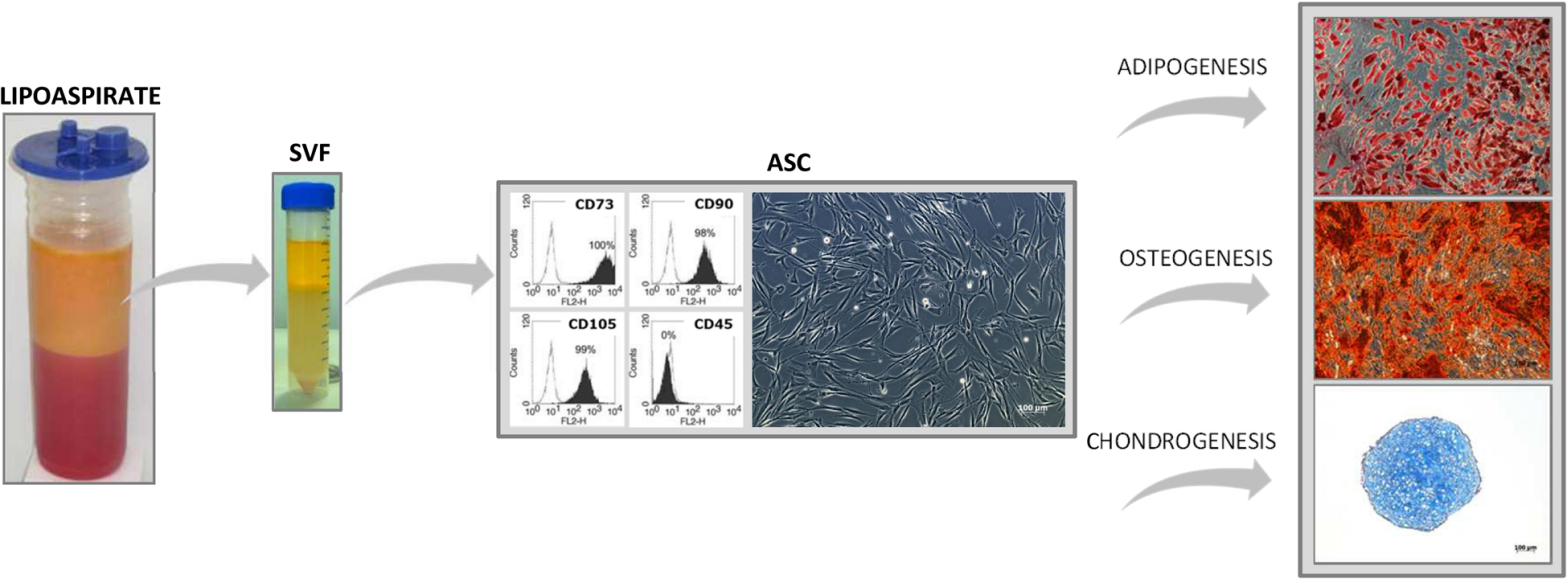
Adipose-derived cells: origin, immunophenotype, morphology, and differentiation potential. Lipoaspirate can be easily obtained from the patient and processed to obtain a heterogeneous cell population, the stromal vascular fraction (SVF). Adipose-derived stromal/stem cells (ASC) can be isolated from the SVF by in vitro cultivation on plastic surfaces. ASC are characterized mainly by mesenchymal stem cell marker (CD73, CD90, CD105) at the expense of hematopoietic stem cell marker (CD45) and their spindle-shaped morphology with the ability to differentiate into the adipogenic, osteogenic, and chondrogenic lineages. The differentiation potential can be analyzed by histological stainings, such as Oil red O for adipogenic, Alizarin red for osteogenic, and Alcian Blue for chondrogenic differentiation

ASC can be isolated from the SVF by in vitro cultivation on plastic surfaces, which results in the accumulation of spindle-shaped cells characterized by their self-renewal potency and ability to give rise to at least adipogenic, osteogenic, and chondrogenic lineages [33–35] (Fig. 1). Besides that, there is a growing body of evidence that these cells can generate a variety of other cell types including neuronal/glial-like cells [36–39], cardiomyocytes [40, 41], endothelial cells [42–44], hepatocyte-like cells [45, 46], various epithelial cell types [47–49], keratinocyte-like cells [50], and dental bud structures [51]. In addition to their extensive differentiation potential, ASC have been shown to secrete high levels of growth factors such as epidermal growth factor (EGF), vascular endothelial growth factor (VEGF), basic fibroblast growth factor (bFGF), keratinocyte growth factor (KGF), platelet-derived growth factor (PDGF), hepatocyte growth factor (HGF), transforming growth factor-beta (TGF-β), insulin growth factor (IGF), and brain-derived neurotrophic factor (BDNF) [52]. The growth factors are secreted at bioactive levels and act primarily angiogenic and anti-apoptotic, and their secretion is significantly increased under hypoxic conditions [53–57]. Besides growth factors, ASC also release cytokines including fms-related tyrosine kinase 3 (Flt-3) ligand, granulocyte-colony stimulating factor (G-CSF), granulocyte macrophage-colony stimulating factor (GM-CSF), macrophage-colony stimulating factor (M-CSF), interleukin (IL) such as IL-6, IL-7, IL-8, IL-11, and IL-12, leukemia inhibitory factor (LIF), and tumor necrosis factor-alpha (TNF-α) [44, 58]. Further, they are able to interact with cells of the immune system and have demonstrated to possess immunomodulatory and anti-inflammatory effects [59–62]. ASC have been successfully used in clinical studies and trials for treating soft tissue defects, bone defects, gastrointestinal lesions, immune disorders, neurological injuries, and cardiovascular diseases [32, 63–72].

### Definition, standardization, and regulation of adipose-derived cells

Although SVF and ASC have been shown to possess a wide therapeutic range in vivo, there is still no standard protocol with uniform parameters for the isolation and also unclear aspects about the identity of cells isolated from adipose tissue. Zuk et al. [2] were the first who identified a cell population derived from human lipoaspirate, which partially resemble BM-MSC in their potency and functionality. These so-called processed lipoaspirate (PLA) cells have the ability to self-renew or differentiate efficiently into the adipogenic, osteogenic, myogenic, and chondrogenic lineage. Various research groups identified consistent immunophenotype surface marker whereas the expression profile changes with passaging. Pittenger et al. [16] identified mesenchymal, hematopoietic, and endothelial stem cell surface marker in BM-MSC. A similar set of markers was identified in SVF and ASC ([34], reviewed in [73]). Castro-Malaspina et al. [74] characterized human MSC within bone marrow via fibroblast colony-forming units showing cell adherence and the ratio between the number of cells plated and the number of colonies formed. Consequently, existing knowledge about SVF and ASC was identified and collected by the International Federation for Adipose Therapeutics and Science (IFATS) together with the International Society for Cellular Therapy (ISCT) in order to provide guidance for standardization between different research groups. IFATS and ISCT established a “living document” for SVF and ASC where existing literature is summarized and can be modified in response to new data and findings from ongoing clinical studies. According to this declaration, cells should have a viability of >70 % for SVF and >90 % for ASC. Frequency of stromal progenitors analyzed with a fibroblast colony forming unit assay (CFU-F) is expected to be >1 % for SVF and >5 % for ASC. Regarding SVF cell identity, immunophenotype should show the following characteristic primary marker profile for stromal cells: CD13, CD29, CD44, CD73, CD90 positive (>40 %), and CD34 positive (>20 %), but CD31 (<20 %) and CD45 negative (<50 %). In contrast, ASC should be positive for CD13, CD29, CD44, CD73, CD90, and CD105 (>80 %), but negative for CD31, CD45, and CD235a (<2 %). Moreover, ASC are expected to have the capacity to differentiate into the adipogenic, osteogenic, and chondrogenic lineage. The differentiation potential can be analyzed by histological stainings, such as Oil red O or Nile red for adipogenic differentiation, Alizarin red or von Kossa for osteogenic differentiation, and Alcian Blue or Safranin O for chondrogenic differentiation. Additionally, specific biomarkers can be investigated, such as adiponectin, CCAAT/enhancer binding protein alpha (C/EBPα), fatty acid binding protein (FABP) 4, leptin and peroxisome proliferator-activated receptor gamma (PPARγ) for adipogenic differentiation, osteocalcin, osterix, and runt-related transcription factor (runx) 2 for osteogenic differentiation and aggrecan, collagen type II, and (SRY (sex determining region Y)-box (Sox) 9 for chondrogenic differentiation [33].

Adipose tissue-derived cells, which are for clinical use, need to fulfill some additional requirements according to current good manufacturing practice (cGMP) compared to those used for research applications. These requirements are set by regulatory agencies and are intended to ensure highest possible safety for patients. A major point is to avoid contact with anything that might carry risk factors, such as pathogens or substances which might cause negative reactions in the patient. This also includes animal-derived components, which are frequently used in common cell culture procedures. Another requirement for production under cGMP is the use of certified and validated instruments and components, which might not be available for every production step. In case of ASC production by common enzymatic method, the following points have to be considered: raw material, in this case adipose tissue, has to be obtained by a validated procedure in a certified facility. This means that the physician’s rooms need to be checked for suitability in addition to applied standard surgery room requirements. Equipment needs to be certified, and personal has to receive appropriate training. To avoid contamination, the graft needs to be transported in a closed sterile container, with suitable temperature maintenance and monitoring. Besides microbiological quality control of the adipose tissue, serological tests of donor blood material have to be performed in order to ensure that no infectious diseases are transferred via the fat graft. A medical questioner’s form has to be answered, and a declaration of consent has to be signed by the donor [75–78].

### Enzymatic and non-enzymatic SVF isolation

The most common isolation technique is based on enzymatic digestion of adipose tissue to obtain the SVF. In general, enzymes such as collagenase, trypsin, or dispase are used to digest adipose tissue [33]. Although the isolation techniques for adipose tissue-derived cells are rather diverse, they follow a certain standard procedure. Differences lie mainly in numbers of washing steps, enzyme concentrations, centrifugation parameters, erythrocyte lysis methods as well as in filtration, and eventually culture conditions [34, 35, 79–85]. Briefly, the adipose tissue is washed, followed by enzymatic digestion and the cells separated by centrifugation from mature adipocytes released oil and enzyme solution. Commonly used collagenases are type I and type II as well as subtypes or combinations of those [26, 82, 85–90]. GMP grade collagenases are produced by recombinant bacteria and are usually delivered in lyophilized form. Their activity varies between batches, and their purity varies between manufacturers. Nevertheless, enzyme concentrations are usually given in weight per volume percent (*w*/*v*) resulting in irregularities between different isolations even if the same protocol is used. Concentrations stated in literature range from 0.075 % (*w*/*v*) to 0.3 % (*w*/*v*) [81, 91, 92]. An erythrocyte lysis step is usually included to get rid of erythrocyte contamination and to decrease the amount of cells with hematopoietic origin. After another optional washing step, the SVF is either cryopreserved or cultured in expansion medium. The plastic-adherent cell fraction, including ASC, can be obtained after passaging or cryopreservation or further cultivated for expansion for a more homogeneous ASC population. As the use of enzymes is accompanied with high costs and might have an impact on safety [79] and efficacy [93, 94], several groups focus on non-enzymatic isolation methods using shear force, centrifugal force, radiation force, and pressure. This mechanical step replaces the enzymatic digestion to separate the cells or cell aggregates from adipose tissue. Nevertheless, similar to the enzymatic cell isolation methods, the range of protocols and methods for the non-enzymatic cell isolation shows also high variations.

### Adipose tissue-derived cell isolation systems

Based on experiences in esthetic surgery, it is known that transfer of autologous fat is beneficial and therefore preferable to all short-acting fillers [95, 96]. But the use of autologous fat can also have serious disadvantages, such as partial necrosis after transplantation of larger quantities. For improving the survival rate of transplanted fat grafts, vascularization is required for nutrition and incorporation inside the surrounding tissue. The additional use of stem cells as SVF and ASC can eliminate these limitations. It has been shown that cell-assisted lipotransfer (CAL) can reduce postoperative atrophy and enhance neovascularization [97–103].

Automated closed devices could help to standardize the isolation process with the assurance of a sterile process. Therefore, a number of different companies together with research laboratories started to develop automated devices to perform parts or even the whole cell isolation process. Some of those devices were primarily designed for enrichment of adipose tissue for autologous fat grafts during plastic surgery and later upgraded for isolation of cell fractions from adipose tissue.

Table 1 summarizes a survey of currently patented, published, or commercially available enzymatic adipose tissue-derived cell isolation systems. The following systems aim for automation of preparation by collagenase-based digestion: AdiStem™ Small/Large Kit and AdiLight (AdiStem Pty Ltd., China), Sepax 2 (Biosafe Group SA, Switzerland), Cellthera Kit I and II and *Method for isolation of adipose tissue-derived stromal vascular fraction* (Cellthera, s.r.o., Czech Republic), A-Stromal™ kit (Cellular Biomedicine Group, Inc./Cellular Biomedicine Group HK, Ltd., USA), Celution® 800/CRS and 820/CRS (Cytori Therapeutics, Inc., USA), Sceldis® (ED Co. Ltd. & Purebiotech Co., Ltd., South Korea/Medica Group, United Arab Emirates), *Automated systems and methods for isolating regenerative cells from adipose tissue* (General Electric Company, USA), GID SVF-1™ (GID Group, Inc., USA), HuriCell (Hurim BioCell, Co., Ltd., South Korea), *Apparatus and methods for cell isolation* (Ingeneron, Inc., USA), STEM-X™ (Medikan International Inc., USA), Beauty Cell (N-Biotek, Inc., South Korea), UNISTATION™ (NeoGenesis Co., Ltd., South Korea), CHA STATION™ and Multi Station (PNC International Co., Ltd., South Korea/PNC North America Division Of Advanced Bio-Medical Equipment Co., INC), CID300 (SNJ Co., Ltd., South Korea/TOPMED CO., LTD., South Korea), Stempeutron™ (Stempeutics Research Pvt. Ltd., India), Tissue Genesis Icellator Cell Isolation System and *Hand-held micro-liposuction adipose harvester, processor, and cell concentrator* (Tissue Genesis, Inc., USA).

**Table 1:** Survey of enzymatic adipose tissue-derived cell isolation systems

Enzymatic SVF isolation systems			
Company	Device/Method	Picture	Publication / Patent
AdiStem Pty Ltd. http://www.adistem.com/technology/adipose-derived-adult-stem-cells/	AdiStem™ Small / Large Kit		[104–107]
	AdiLight		
Biosafe Group SA http://www.biosafe.ch/	Sepax 2		[86]
Cellthera, s.r.o. http://www.cellthera.org	Cellthera Kit I and II *Method for isolation of adipose tissue-derived stromal vascular fraction cells*		[32, 141]
Cellular Biomedicine Group, Inc./Cellular Biomedicine Group HK, Ltd. http://cellbiomedgroup.com/newsroom/new-product-new-license/	A-Stromal™ kit		-
Cytori Therapeutics, Inc. http://www.cytori.com	Celution® 800/CRS		[87, 108–111, 120, 142–146]
ED Co. Ltd. & Purebiotech Co., Ltd./Medica Group	Sceldis®	–	[114, 115]
http://www.medicagroup.com/Sceldis			
General Electric Company	*Automated systems and methods for isolating regenerative cells from adipose tissue*	–	[116]
-			
GID Group, Inc.	GID SVF-1™	–	[113, 147, 148]
http://www.thegidgroup.com/			
http://www.gideurope.com/gid-system/			
Hurim BioCell, Co., Ltd.	HuriCell	–	[117, 149]
http://a-swiss.org/aestetics/huricell/			
Ingeneron, Inc. -	*Apparatus and Methods for Cell Isolation*		[118]
			
Medikan International Inc. http://doc.xueqiu.com/144d468295a2f23f9789fc75.pdf	STEM-X^TM^		-
N-Biotek, Inc.	Beauty Cell	–	-
http://n-biotek.com			
NeoGenesis Co., Ltd. http://eng.neogenesis.co.kr/	UNISTATION™		-
PNC International Co., Ltd. / PNC North America	CHA STATION™	–	[108]
Division Of Advanced Bio-Medical Equipment Co., INC	Multi Station		
http://www.pncint.com/			
http://pnc-na.com/			
SNJ Co., Ltd. / TOPMED CO., LTD.	CID300	–	-
http://www.globalsources.com/si/AS/SNJ-Co/6008829321267/pdtl/Full-automatic-High-Yield-Rate/1118766725.htm			
Stempeutics Research Pvt. Ltd.	Stempeutron™	–	[150]
http://www.stempeutics.com/stempeutron.html			
Tissue Genesis, Inc.	Tissue Genesis^®^ Icellator Cell Isolation System™	–	[119, 151–153]
http://www.tissuegenesis.com/icellator.html	*Hand-held micro-liposuction adipose harvester, processor, and cell concentrator*	–	

Patented, published or commercial available systems are listed with the associated company (in alphabetical order), a picture of the system as well as links and references if accessible. The majority of the systems generates stromal vascular fraction (SVF). (Italic represents the title of patents, − not available)

Other systems do not include enzymatic digestion but break the processed adipose tissue by mechanical forces. Table 2 summarizes a survey of currently patented, published, or commercially available non-enzymatic adipose tissue-derived cell isolation systems. The following non-enzymatic cell isolation systems generate SVF-enriched adipose tissue: *Devices for harvesting and homogenizing adipose tissue containing autologous endothelial cells* (Baxter International Inc., USA), Puregraft® (Bimini Technologies LLC, USA), Fastkit (Fastem) (CORIOS Soc. Coop., Italy), LipiVage™ (Genesis Biosystems, Inc., USA), Revolve™/GID 700™ (LifeCell Corporation, USA/GID Group, Inc., USA), Lipogems® (Lipogems International S.p.A., Italy), Lipo-Kit GT (Medikan International Inc., USA), StromaCell™ (MicroAire Surgical Instruments, LLC, USA), and myStem® (MyStem LLC, USA). Several other non-enzymatic isolation systems aim at the isolation of adipose tissue-derived cells and obtain pure SVF: *Method for isolating stromal vascular fraction* (Agency Science, Tech & Res, China), *Procedure and device for separating adult stem cells from fatty tissue* and *Device for separating adult stem cells* (Human Med AG, Germany), *Ultrasonic cavitation derived stromal or mesenchymal vascular extracts and cells derived therefrom obtained from adipose tissue and use thereof* and *Isolation of stromal vascular fraction from vascular tissues* (IntelliCell BioSciences Inc., USA), *Non-enzymatic method for isolating human adipose-derived stromal/stem cells* (Pennington Biomedical Research Center, USA), *Isolation of stem cells from adipose tissue by ultrasonic cavitation, and methods of use* (Rusty Property Holdings Pty Ltd., Australia/Amberdale Enterprises Pty Ltd., Australia/Tavid Pty, Australia), and *Selective lysing of cells using ultrasound* (Solta Medical, Inc., USA).

**Table 2:** Survey of non-enzymatic adipose tissue-derived cell isolation systems

Non-Enzymatic SVF isolation systems			
Company	Device/Method	Picture	Publication / Patent
Agency Science, Tech & Res	*Method for isolating stromal vascular fraction*	–	[154]
-			
Baxter International Inc.	*Devices for harvesting and homogenizing adipose tissue containing autologous endothelial cells*	–	[135]
-			
Bimini Technologies LLC -	Puregraft®		[121, 122]
CORIOS Soc. Coop. http://www.corios.it	Fastkit (Fastem)		[120]
Genesis Biosystems Inc. http://genesisbiosystems.com/products/lipivage/	LipiVage™		[125, 126, 155]
Human Med AG	*Procedure and device for separating adult stem cells from fatty tissue*	–	[156]
-	*Device for separating adult stem cells*	–	[157, 158]
IntelliCell^®^ BioSciences Inc.	*Ultrasonic cavitation derived stromal or mesenchymal vascular extracts and cells derived therefrom obtained from adipose tissue and use thereof*		[159]
http://www.intellicellbiosciences.com	*Isolation of stromal vascular fraction from vascular tissues*		[136, 160]
LifeCell Corporation / GID Group, Inc.	Revolve™ / GID 700™	–	[123, 124, 161, 162]
http://www.lifecell.com/			
http://www.thegidgroup.com/			
http://www.gideurope.com/gid-system/			
Lipogems International S.p.A. http://www.lipogems.eu/	Lipogems^®^		[127–134, 163]
Medikan International Inc. http://www.medikanint.com/	Lipo-Kit GT		[108, 120]
MicroAire Surgical Instruments, LLC	StromaCell™	–	[164]
http://www.microaire.com			
MyStem LLC http://www.mystem.info	myStem^®^		-
Pennington Biomedical Research Center	*Non-Enzymatic Method for Isolating Human Adipose-Derived Stromal Stem Cells*	–	[140, 165]
-			
Rusty Property Holdings Pty Ltd, / Amberdale Enterprises Pty Ltd, / Tavid Pty	*Isolation of stem cells from adipose tissue by ultrasonic cavitation, and methods of use*	*	[137, 138]
-			
Solta Medical, Inc.	*Selective lysing of cells using ultrasound*	–	[139]
-			

Patented, published or commercial available systems are listed with the associated company (in alphabetical order), a picture of the system as well as links and references if accessible. The majority of the systems generates cell enriched adipose tissue, while some systems obtain stromal vascular fraction (SVF). (Italic represents the title of patents, * currently upgrading technology, − not available)

Note: Affiliations are status May–July 2015. Due to the fast growing/changing market, some of the device/company names might have already changed at time of publication.

### In vitro/in vivo analyses of cells derived from enzymatic isolation devices/procedures

Several of the presented systems have already been tested in preclinical and clinical studies, and few comparative studies using cells isolated by different systems. SVF cells isolated using AdiStem™ cell isolation kit were combined with platelet-rich plasma (PRP) and transplanted into nude NOD/SCID mice resulting in joint regeneration [104]. AdiStem™ isolated cells were activated with a photobiostimulator AdiLight with a total viable cell number of 12 × 10^6^/ml fat compared to a standard isolation method with 10 × 10^6^ cells/ml fat [105]. AdiLight photobiostimulation-activated SVF were endobronchially infused to idiopathic pulmonary fibrosis (IPF) patients with an output of marginal improvement of walking and forced vital capacity [106]. Another safety study with this system was performed using infusions of autologous SVF in IPF patients with no deterioration in functional parameters and quality of life [107]. A major study was performed by Michalek et al. [32] where 1128 patients suffering from osteoarthritis were treated with autologous SVF cells isolated with Cellthera Kit I and II. Patients observed an improvement in pain, movement, and stiffness after SVF injection directly into the joint. Enzymatic cell isolation using the Celution® 800/CRS system exhibits a cell number of 2.95 × 10^5^ cells/ml with a viability of 86.6 % [87]. In a comparison test, the Celution® 800/CRS system demonstrated the highest cell yield (2.41 × 10^5^ cells/g) compared to Multi Station (1.07 × 10^5^ cells/g), Lipo-Kit GT (0.35 × 10^5^ cells/g), and CHA STATION™ (0.05 × 10^5^ cells/g) [108]. Adipose-derived regenerative cell (ADRC)-enriched fat grafting using the Celution® 800/CRS device resulted in decreased fat absorption and increased neovascularization in nude mice [109]. Autologous SVF cells isolated with the same device improved hand disability and reduced pain in systemic sclerosis [110]. With the same system, autologous ADRC and adipose tissue were transurethrally injected, resulting in reduction of male stress urinary incontinence [111]. ADRC-enriched fat graft injections derived from Celution® device improved breast contour in a clinical trial for breast conservation therapy (BCT) [112]. Isolation of SVF with the GID SVF-1™ yielded an average cell number of 7.19 ± 2.11 × 10^5^ nucleated cells/ml of dry adipose tissue. This cell number is dependent on the patient’s age and decreases with increasing age [113]. Güven et al. [86] observed a higher cell yield with the Sepax 2 isolation system compared to standard manual isolation (2.6 ± 1.2 × 10^5^ vs. 1.6 ± 0.9 × 10^5^ nucleated cells/ml of liposuction) and 24 % higher clonogenicity. SVF cells derived via Sceldis® were added to a mixture of platelet-rich plasma, hyaluronic acid, and CaCl_2_ and injected to treat knee pain due to meniscus tear [114, 115]. The invention of Khan et al. resulted in cell numbers of ~6 × 10^5^ cells/ml (66 % viability) for donor 1 and ~1 × 10^6^ cells/ml (51 % viability) for donor 2 [116]. In an animal model of focal cerebral ischemia, cells isolated with the HuriCell isolation device showed neuroprotective effects in ischemic brain injury [117]. Stubbers and Coleman [118] invented an apparatus and methods for cell isolation yielding 4.9 × 10^6^–24.7 × 10^6^ total nucleated cells/100 g lipoaspirate. SVF isolated with the Tissue Genesis Icellator Cell Isolation System were successfully used with aspirated adipose tissue for breast and face augmentation or reconstruction [119]. Domenis et al. [120] analyzed three isolation devices (Lipo-Kit, Celution®, Fastem) and showed that SVF-enriched adipose fat grafts increased thickness of the tissue and improved long-term effects in breast reconstruction compared to standard lipotransfer. However, the non-enzymatic device Fastem was less effective in stem cell enrichment compared to the enzymatic devices Lipo-Kit and Celution®.

### In vitro/in vivo analyses of cells derived from non-enzymatic isolation devices/procedures

Washing and filtration of adipose tissue using Puregraft® showed higher tissue viability and lower presence of red blood cells, free lipids, and contaminants compared to other fat grafts [121, 122]. Processed adipose tissue derived from the Revolve™ system exhibited significant higher fat retention injected in nude mice than with a standard centrifugation or decantation method [123]. Washing of adipose tissue with the GID 700™ resulted in significant reduced amounts of triglycerides, lactate dehydrogenase, and hematocrit and maintained osmolarity of the adipose graft [124]. SVF cells derived from the harvesting and irrigation device LipiVage™ showed mesenchymal and endothelial progenitor cells maintaining their growth and differentiation capacity when applied through a fibrin spray system [125]. LipiVage™ yielded a higher number of viable adipocytes and sustained a higher level of intracellular enzyme (glycerol-3-phophatase dehydrogenase (G3PDH)) activity within fat grafts [126]. Bianchi et al. [127] showed that Lipogems® isolated cells exhibit a significantly higher percentage of mature pericytes and ASC and lower amount of hematopoietic cells than enzymatic isolated cells and higher percentage of exosomes [128]. Moreover, Lipogems® showed arteriogenic and paracrine properties for the rescue of ischemic limb [129] and ASC derived from Lipogems® exhibit enhanced transcription of vasculogenic genes, enhanced differentiation capacity in mouse embryonic stem cells, and efficient direct multi-lineage reprogramming in human skin fibroblasts compared to enzymatically isolated ASC when exposed to a radio electric asymmetric conveyer (REAC) [130]. Human lipoaspirated adipose tissue microfragmented with Lipogems® resulted in a better mesenchymal stem cell source compared to normal lipoaspirated tissue, while maintaining the structural composition of the original tissue [131]. Fat transplantation using Lipogems® applied in combination with orthognatic surgery reduces postoperative pain and swelling and improves final esthetic outcomes [132]. In addition, Lipogems® may improve the healing, osteointegration, and stability of the implants in newly formed bone [133] and can also improve the symptoms of fecal incontinence due to muscular and neural local trauma [134]. Another device for harvesting and homogenizing adipose tissue for endothelial cells was described by Hu et al. [135] obtaining 1.12–2.13 × 10^6^ cells larger than 7.8 μm from 1 g adipose tissue but after enzymatic isolation of the non-enzymatic isolated cells. An increased population of microvascular endothelial cells can be collected with an elongated cannula with cutting edges to disrupt the connective tissue. Victor invented a SVF isolation method using ultrasonic cavitation and yielded 1.67–2.24 × 10^7^ cells with a viability of 97.1–98.9 % [136]. Bright et al. [137] dissociated adipose tissue by lysing mature adipocytes using ultrasonic cavitation to obtain cell yields of about 2–4 million cells/gram adipose tissue. Clinical studies were performed using intra-articular SVF injection for patients suffering from osteoarthritis (knee, hip) and intravenous SVF injection for rheumatoid osteoarthritis showing improvement in pain, stiffness, and physical function. Patients suffering from chronic migraine experienced a decline in frequency and severity of migraines after systemic treatment with autologous SVF isolated with the ultrasonic cavitation protocol from Bright et al. [138]. Another method was claimed by Schafer [139], focusing on the separation of adipose cells using ultrasonic energy/acoustic standing wave with a yield of small (<50 μm) but more vital pre-adipocytes for successful grafting. This graft could stimulate the production of vascular structure. The idea of a simple method comprising manual shaking and washing the stem cells out from adipose tissue derives from Gimble et al. [140] with a cell yield of 2.5 × 10^6^ cells per 100 ml adipose tissue. These cells possess equally high adipogenic and osteogenic differentiation potential, compared to a standard enzymatic isolation method.

## Conclusion

In the past decade, subcutaneous adipose tissue came into the focus of plastic surgery and regenerative medicine. The isolated SVF as well as ASC have been successfully used in clinical studies and trials. But there are still drawbacks associated with current strategies to provide cellular therapeutics, which is defined in the regulations of the different countries. To fulfill the criteria of the regulatory authorities for the translation of cell-based therapies into clinics, a great deal of work remains to be done: primarily, a common standard operating protocol, toxin- and xeno-free reagents including replacement of enzymes and a quick quality control to predict donor variations in cell identity and efficiency. Therefore, several adipose tissue-derived cell isolation systems have been already developed with the main goal to provide a closed, sterile, and safe isolation process avoiding contaminations and unpredictability of the cell material. However, not all of the cell isolation systems are closed systems, which is the prerequisite for a sterile isolation unless the isolation is performed in a cleanroom facility. Each method or system has different advantages and disadvantages and is under continuous development and optimization. Differences within the systems include parameters such as operation (manual or automatic), handling (easy until cumbersome to use), and costs (e.g., expensive apparatus or high cost consumables). Publication of study outcomes, comparative studies as well as standardization of cell products will allow the field to bring further effective therapy to the clinics.
